# Surgeon General’s Report

**Published:** 1869-02

**Authors:** J. K. Barnes

**Affiliations:** Surgeon Gen’l, U. S. Army


### Surgeon General's Report.

We copy nearly entire the Surgeon General's Report, omitting only the "*financial statement*":

ANNUAL REPORT.

*War Department, Surgeon General's Office,*
Washington, D. C., October 20, 1868.

Hon. JOHN M. SCHOFIELD, Secretary of War:

Sir:—I have the honor to submit the following statement of finances and general transactions of the Medical Department of the Army for the fiscal year ending July 1, 1868:

At the date of my last Annual Report, Epidemic Cholera and Yellow Fever prevailed among the troops in various sections of the country, a very full and exhaustive report of which was published for the information of medical officers of the Army in Circular No. 1, War Department, Surgeon General's Office, June 10, 1868. To this date, there has been no well authenticated case of epidemic cholera or of yellow fever reported as occurring among troops in the present year.

The monthly reports of sick and wounded for the fiscal year terminating June 30, 1868, received in the division of records of this office to this date, represent an average mean strength of forty-five thousand two hundred and fifty-seven white, and four thousand seven hundred and seventy-four colored, troops.

For the *white troops*, the total number of cases of all kinds reported under treatment was one hundred and thirty-one thousand five hundred and eighty-one, or two thousand nine hundred and eight per thousand of strength—nearly three entries on the sick report during the year for each man. Of this number of cases, one hundred and eighteen thousand nine hundred and twenty-five were for disease alone, and twelve thousand six hundred and fifty-six for wounds, accidents and injuries; being two thousand six hundred and twenty-eight per thousand of strength for disease, and two hundred and eighty per thousand of strength for wounds, accidents and injuries. The average number constantly on sick report was two thousand eight hundred and fifty-two, of whom two thousand five hundred and ten were sick and three hundred and forty-two wounded, or fifty-five per thousand constantly under treatment for disease and eight per thousand for wounds and injuries. The total number of deaths from all causes reported, was one thousand three hundred and fifty-three; of which, one thousand one hundred and seventy-five were from disease, and one hundred and seventy-eight for wounds, accidents and injuries; being at the rate of twenty-six deaths from disease and four from wounds to each thousand of strength. Of the deaths from disease, four hundred and twenty-seven were from yellow fever, one hundred and thirty-nine from cholera, and six hundred and nine, or thirteen deaths per thousand of strength, from all other diseases. The proportion of deaths from all causes to cases treated was one death to ninety-seven cases.

Nine hundred and eighty-four white soldiers, or twenty-two per thousand of strength, were discharged upon Surgeon's certificate of disability.

For the *colored troops*, the whole number of cases of all kinds treated was fourteen thousand six hundred and sixteen; being at the rate of three thousand and sixty-one per thousand of strength, or three cases of sickness for each man. Of this number, thirteen thousand five hundred and fifty were for disease; being two thousand eight hundred and thirty-eight per thousand of strength; one thousand and sixty-six were for wounds, accidents and injuries; being two thousand and twenty-three per thousand. The average number constantly on sick report was two hundred and eighty-three; of whom two hundred and forty-eight were sick and thirty-five wounded; being at the rate of fifty-two per thousand constantly under treatment for disease, and seven per thousand for wounds, accidents and injuries.

The total number of deaths reported was two hundred and sixty-eight; of which two hundred and forty-two were from disease, twenty-six from wounds and injuries; being at the rate of fifty-one deaths per thousand of strength from disease, and five per thousand from wounds. Of the deaths from disease, twenty-five were from yellow fever, eighty-nine from cholera; leaving one hundred and twenty-eight, or twenty-seven per thousand of strength, from all other diseases. The proportion of deaths from all causes to cases treated was one death to fifty-five cases.

Ninety colored soldiers, or nineteen per thousand of strength were discharged on Surgeon's certificate of disability.

During the year, the records filed in the record and pension division of this office have been searched, and such official information relative to deaths, discharges and treatment as they contain has been furnished in reply to the inquiries of the Pension Bureau, in sixteen thousand seven hundred and eighty-six cases; Adjutant General, U. S. A., in fifteen thousand five hundred and eighty-two cases; Paymaster General, U. S. A., in four hundred and seventy-three cases; and in nineteen hundred and twenty-nine cases to other authorized inquirers, making a total of thirty-four thousand seven hundred and seventy.

In the division of surgical records the histories of seventy-four thousand nine hundred and fifty-four cases of wounds and injuries have been transcribed, chiefly from field reports, hospital case books and registers of 1861 and 1862 and the earlier part of 1863.

The records of the office in regard to injuries of the head, face, neck, thorax, abdomen, spine and pelvis have been classified and studied. Illustrative cases have been selected and written out in minute detail, while numerical tables have been prepared, exhibiting the progress and results of the different classes of injuries to which these individual examples belong. To illustrate these injuries, for future publication, there have been completed during the year, eight chromo-lithographs, eight lithographs and three diagrams. There have also been prepared during the year one hundred and twenty-two wood-cuts, to be intercalated in the text descriptive of the various classes of injuries and operations. Five hundred pages of manuscript are in readiness for the printer, and a large amount of the statistical material is in such state of forwardness that it can be made ready for the press at a few weeks' notice. To make the publications of this office as valuable as possible, in relation to the results of the major surgical injuries and operations, and especially in regard to the excisions of the larger joints and other operations embraced under the general designation of conservative surgery, much time and labor have been expended in tracing the ultimate histories of patients who have undergone such mutilations. This has been accomplished to a very satisfactory degree, through the operations of the examining surgeons of the Pension Bureau, of the Surgeons General and Adjutants General of the several States, of retired volunteer medical officers, and of private physicians. Besides the digestion and tabulation of the surgical data pertaining to the late war, there have been received and consolidated, six hundred and ninety-nine quarterly reports of post hospitals, thirty-four reports of the examination of men, who, having been wounded, presented themselves for re-enlistment at recruiting stations, and thirty-two special reports of surgical operations.

The Army Medical Museum continues to increase in value and usefulness. During the year, six hundred and seventy-three specimens have been added to the surgical section, one hundred and twenty-one to the medical section, two hundred and two to the section of comparative anatomy, six hundred and eighty-seven specimens and one hundred and fourteen photographic negatives of microscopical specimens to the microscopical section. An anatomical section of one hundred and sixty-three specimens has been formed, and is rendered of especial interest by the large proportion of typical crania of the North American Aborigines which it contains. A collection of one hundred and eighty-seven specimens of Indian weapons and utensils has also been added. Two hundred and sixty-six discarded specimens, the histories of which could not be found at the period of publication of the Catalogue of the Surgical Section, have been identified and restored to the collection. For the purpose of exchange with other museums, or with learned societies, either for specimens or publications, four thousand four hundred and seventy-two photographs, illustrative of injuries and operations, have been printed. There were during the year fourteen thousand four hundred and forty-eight visitors to the Museum, including many military surgeons of eminence.

On the 30th of September there were two hundred and eighty-nine garrisoned posts in the various military departments, besides an almost equal number of detachments on temporary duty throughout the South and on expeditions, or protecting the lines of travel on the plains, requiring medical attendance. The number of Surgeons and Assistant Surgeons being altogether inadequate to meet this demand, it has been necessary to employ contract physicians, especially at the South, where but few of the resident physicians could take the oath necessary to their payment, and the fees for attendance in individual cases would be far in excess of the contract rates. The number of physicians so employed upon the 30th of September was two hundred and eighty-two, at rates of compensation varying from $45.00 to $125.00 per month; but a large proportion of these will be dispensed with so soon as the troops are concentrated in winter quarters and the condition of public affairs will admit of the discontinuance of the numerous small garrisons throughout the States recently in rebellion.

Since the date of my last Annual Report, three Surgeons and two Assistant Surgeons have died, eight Assistant Surgeons have resigned, two Assistant Surgeons have been dismissed, and one Assistant Surgeon cashiered. Total, sixteen.

A Medical Board, for the examination of candidates for appointment as Assistant Surgeons, U. S. Army, and of Assistant Surgeons for promotion, is now in session in New York city,

There are now forty-nine vacancies in the grade of Assistant Surgeon.

Most respectfully, your obedient servant,

J. K. Barnes,

Surgeon Gen'l, U. S. Army.

